# Lipotoxicity in Kidney, Heart, and Skeletal Muscle Dysfunction

**DOI:** 10.3390/nu11071664

**Published:** 2019-07-20

**Authors:** Hiroshi Nishi, Takaaki Higashihara, Reiko Inagi

**Affiliations:** 1 Division of Nephrology and Endocrinology, the University of Tokyo Graduate School of Medicine, Tokyo 113-8655, Japan; 2 Division of CKD Pathophysiology, the University of Tokyo Graduate School of Medicine, Tokyo 113-8655, Japan

**Keywords:** lipotoxicity, kidney, uremia, skeletal muscle, sarcopenia

## Abstract

Dyslipidemia is a common nutritional and metabolic disorder in patients with chronic kidney disease. Accumulating evidence supports the hypothesis that prolonged metabolic imbalance of lipids leads to ectopic fat distribution in the peripheral organs (lipotoxicity), including the kidney, heart, and skeletal muscle, which accelerates peripheral inflammation and afflictions. Thus, lipotoxicity may partly explain progression of renal dysfunction and even extrarenal complications, including renal anemia, heart failure, and sarcopenia. Additionally, endoplasmic reticulum stress activated by the unfolded protein response pathway plays a pivotal role in lipotoxicity by modulating the expression of key enzymes in lipid synthesis and oxidation. Here, we review the molecular mechanisms underlying lipid deposition and resultant tissue damage in the kidney, heart, and skeletal muscle, with the goal of illuminating the nutritional aspects of these pathologies.

## 1. Basic Biochemistry of Lipids and Fatty Acids

Lipids are a heterogeneous group of hydrophobic organic molecules that are extracted from tissues and nonpolar solvents. Because of their insolubility in water, biological lipids are categorized by structures, including membrane-associated lipids, droplets of triacylglycerol (TAG) in adipose tissue, and bound to albumin during transportation in plasma. Fatty acids (FAs) with 12 carbons or more are referred to as long-chain FAs (LCFAs), while FAs with less than 12 carbons are referred to as short-(SCFAs) and medium-chain FAs (MCFAs). FA chains may contain no double bonds (saturated) or one or more double bonds (unsaturated). In standard nomenclature, FAs are identified by two numbers separated by a colon, followed by a number(s) in parentheses; the number before the colon indicates the number of carbons in the chain, and the number after the colon indicates the number of double bonds. For example, palmitic acid (PA), 16:0, is a representative saturated FA (SFA) abundant in the body, while oleic acid (OA), 18:1, is an unsaturated FA (USFA). Theoretically, the yield from complete oxidation of FAs to CO_2_ and H_2_O_2_ is 9 kcal/g fat, while it is only 4 kcal/g for protein and carbohydrate.

In a well-fed state, TAG contained in chylomicrons is degraded to FA and glycerol by lipoprotein lipase and stored as TAG in the capillaries of the kidney, as well as the heart, skeletal muscle, and adipose tissue.

**Figure 1:**
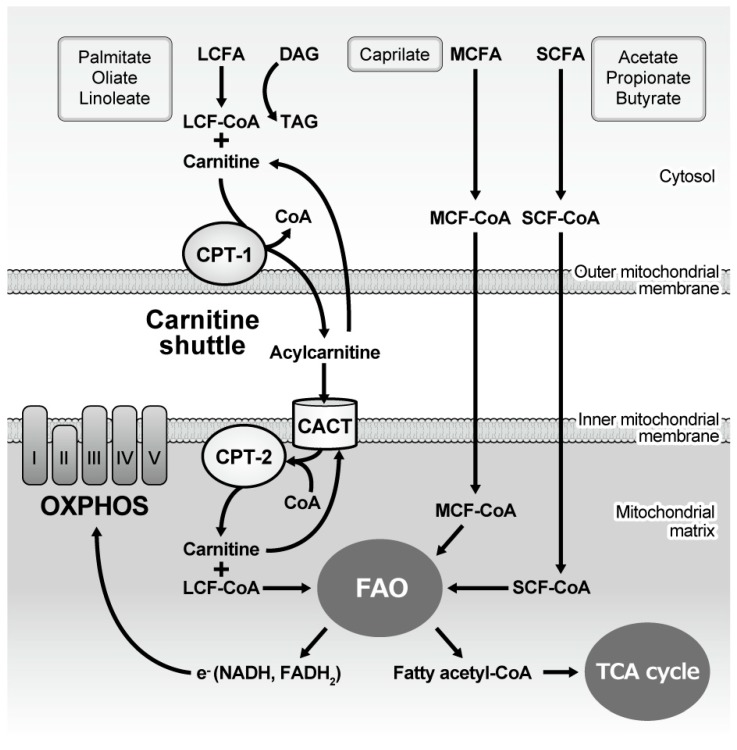
Mitochondrial handling of fatty acids and subsequent energy generation. Long-chain fatty acyl-CoA is esterified with carnitine in the cytoplasm and then the carnitine shuttle serves to carry it from the cytosol to the mitochondria, while medium- and short-chain fatty acyl-CoA freely diffuses into the mitochondria. Once the fatty acid is located inside the mitochondrial matrix, two carbons are cleaved from the molecule every cycle to form fatty acetyl-CoA, the process of which is named fatty acid oxidation (β oxidation). The process continues until all of the carbons in the fatty acid are turned into acetyl CoA, which enters the TCA cycle to generate ATP. Oxidation also generates NADH and FADH_2_, electrons derived from which are utilized by the five OXPHOS complexes to generate ATP. CPT: Carnitine palmitoyltransferase; CACT: Carnitine-acylcarnitine translocase; CoA: Coenzyme A; LCFA: Long-chain fatty acid; MCFA: Medium-chain fatty acid; SCFA: Short-chain fatty acid; FAO: Fatty acid oxidation; TCA: Tricarboxylic acid; NAD: Nicotinamide adenine dinucleotide; FAD: Flavin adenine dinucleotide; OXPHOS: Oxidative phosphorylation.

In contrast, during caloric deficiency, hormone-sensitive lipase, which is activated by epinephrine and glucagon, degrades stored TAG in adipose cells. FAs are transported by serum albumin to the liver and periphery. Then, in the cytosol, FA is esterified to fatty acid acyl coenzyme A (FA-CoA) (Figure 1). The carnitine shuttle, mediated by carnitine palmitoyltransferase I (CPT1) and II (CPT2), serves to carry LCFAs from the cytosol to the mitochondria in a rate-limiting process. LCA-CoAs are primarily converted to acylcarnitine by CPT, which facilitates this shuttle. However, SCFA and MCFA can cross the inner mitochondrial membrane without this machinery. In the mitochondria, FA degradation (β-oxidation) eventually occurs as two-carbon fragments are successively removed from the carboxyl end of the fatty acyl CoA, producing fatty acetyl CoA, NADH, and FADH_2_. The majority of fatty acetyl CoA enters TCA to produce ATP, while electrons released from NADH and FADH2 are utilized in the electron transport chain in the mitochondrial inner membrane (Figure 1).

## 2. Nutritional Aspect of Lipids

During initial digestion of dietary lipids, they are emulsified in the gastrointestinal tract because of their hydrophobicity. For example, TAGs in dairy products contain SCFA and MCFA that are principally digested in the stomach. In contrast, LCFA contained in cholesteryl esters (CE), phospholipids, and TAG, should be degraded in the intestine. The resultant products are 2-monoacylglycerol, unesterified cholesterol, and free FA. The intestinal epithelium absorbs these dietary lipids as mixed micelles; resynthesizes TAG, CE, and phospholipids; synthesizes apolipoprotein B-48; and eventually assembles chylomicrons.

One of the general discussion topics regarding diet and energy expenditure is whether a diet high in fat or a diet high in carbohydrates is beneficial for controlling lipid metabolism and body weight. The carbohydrate-insulin model of obesity [1] verifies the logic that the increased ratio of insulin to glucagon concentration after consuming a diet with a high glycemic load directs metabolic fuels from consumption toward storage in adipose tissue. However, this dogma has been challenged because evidence from controlled studies is lacking [2]. Indeed, a recent meta-analysis reported no meaningful difference in energy expenditure between low carbohydrate and low fat diets [3]. Recently, Ebbeling et al. compared the effects of diets varying in carbohydrate to fat ratios on energy expenditure and demonstrated by intention-to-treat analysis that high fat/low carbohydrate diet increased energy expenditure during weight loss maintenance [4]. The authors provided evidence supporting that processed carbohydrates negatively effect metabolism; high fat content in lower-carbohydrate or ketogenic (very low-carbohydrate) diets are better for health and that not the relative quantity of dietary fat and carbohydrate is less important than the type of fat or carbohydrate source [5].

Dietary protein affects lipotoxicity. In this respect, Tovar et al. found that the type of dietary protein determines the process of ectopic lipid deposition by modulating insulin secretion and regulating adipocyte metabolic function [6]. For example, compared to casein, soy protein and its isoflavones only mildly promote insulin secretion but enhance insulin sensitivity by lowering sterol regulatory element-binding protein-1 (SREBP-1) expression in the liver, leading to low accumulation of hepatic TG and lipid deposits and ceramide. Sirtorim et al. showed that bioactive peptide(s) can may be able to be discovered from both vegetable and animal sources to reduce human blood cholesterol level [7]. Intriguingly, Inoue et al. identified several soy protein-derived hypotriglyceridemic di-peptides [8]. Recent papers have tested the positive effects of natural compounds in liver steatosis, which are promising as lipid-lowering drugs [9,10,11].

## 3. Lipotoxicity as an Emerging Pathogenic Entity

Lipotoxicity is defined as the accumulation of lipid intermediates and final products in tissues and organs in which it typically does not accumulate. Lipotoxicity is thought to play important roles in heart failure, obesity, and diabetes, and is estimated to affect approximately 25% of the adult US population [12]. Those normally affected include the kidneys, liver, heart, and skeletal muscle. The effects of fat accumulation have been well-studied in the pancreas and liver [9,10]. These ectopic fat deposits may act as active machinery, releasing a variety of in vivo biochemical mediators that influence insulin resistance and inflammation, both of which enhance cardiovascular risk [13]. In pancreatic β-cells, for example, excess FA negatively affects the synthesis, secretion, and function of insulin, exacerbating glucose intolerance. Glycometabolic disorders occur less frequently, as FAs are delivered by systemic circulation to the liver, pancreatic β-cells, and skeletal muscles [13].

**Figure 2:**
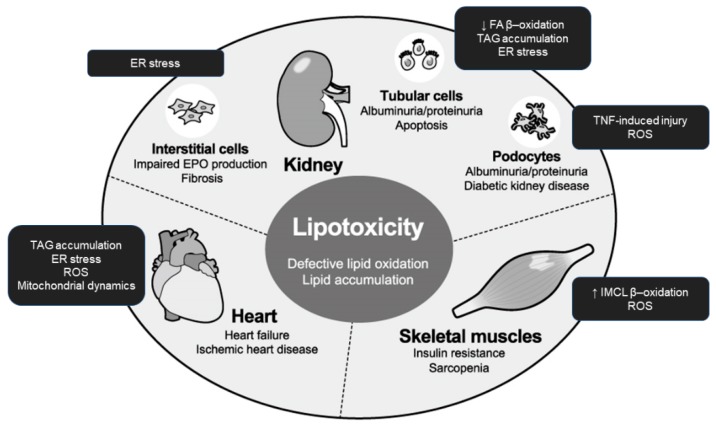
Integrated organ pathology caused by lipotoxicity in uremia. In the presence of prolonged nutrient excess or disturbed metabolism, lipids accumulate ectopically in tissues and organs including the kidney, heart, and skeletal muscle. The endpoints of lipotoxicity vary among those targets and include urinary albumin excretion and impaired erythropoietin production in the kidney, heart failure, and insulin resistance and mass reduction (sarcopenia) in the skeletal muscle. EPO: erythropoietin; ER: endoplasmic reticulum; ROS: reactive oxygen species; FA: fatty acid; TNF: tumor necrosis factor; IMCL: intramyocellular lipid; TAG: triacylglycerol.

In this review, we focus on lipotoxicity in the kidney, heart, and skeletal muscles during kidney disease progression, which have been less emphasized as lipotoxicity target organs but associated with each other (Figure 2). Kidney dysfunction over a period of months or years is known as chronic kidney disease (CKD). CKD is growing global health burden and leads to end-stage kidney disease (ESKD) requiring renal replacement therapy such as transplantation or dialysis, but also causes dysfunction and disorders in extra-renal tissues and organs. Anemia, defined as a lowered hemoglobin level in the bloodstream due to impaired production of red blood cells, commonly occurs in people with CKD. Congestive heart failure (CHF) or simply heart failure (HF), defined as a chronic and progressive condition in which the heart muscle is unable to pump enough blood through the heart to meet the body’s needs, is also common in CKD patients. Because of its vitality, fatal comorbidity in CKD patients comprises heart issues including CHF, myocardial infarction, cardiac arrhythmia, and sudden death. Additonally, skeletal muscle is a less-recognized afflicted organ in CKD. Skeletal muscle is an important endocrine organ in terms of glucose metabolism, and insulin resistance is an early metabolic alteration in patients with CKD. Uremic sarcopenia, defined as skeletal muscular mass reduction and dysfunction, is skeletal complication of CKD associated with poor life activity and prognosis.

The common mechanism underlying multiple tissue and organ damage in the uremic milieu has been a target of intensive clinical and experimental research for numerous years. Although hypertension, malnutrition, and uremic toxins may be contributory, it is noteworthy that dyslipidemia (DL) shows a high clinical prevalence in patients with CKD and significant alterations in lipoprotein metabolism [14,15]. Dysregulated lipid metabolism and the resultant lipotoxicity in end-organs may explain why multiple organs are afflicted in parallel during CKD progression. Another reason to focus on lipotoxicity in CKD is that diabetic nephropathy (DN) and diabetic kidney disease (DKD) constitute nearly half of ESKD cases. Thus, diabetes progression is closely related to metabolic syndrome and obesity, which involve not merely an expansion of the adipose tissue mass, but also includes the activation of inflammatory processes within the visceral adipose tissue as well as skeletal muscle, heart, skeletal muscle, pancreas, and liver [16,17]. Thus, in the context of lipotoxicity, fatty acid derivatives can ultimately lead to gradual organ failure. This can occur in patients with CKD as the prevalence of obesity in patients with ESKD increased [18].

We review how lipotoxicity impairs physiological function and causes not only renal damage but also cardiac and skeletal muscular complications.

## 4. Lipotoxicity in the Kidney

The kidney has a complex architecture comprised of multiple, highly specialized cell types. Given the high volume of blood that passes it through, this organ is easily affected by the amount and quality of circulating FA. Indeed, the kidney is negatively affected by DL, lipid accumulation, and changes in adipokines in the bloodstream that change peripheral lipid metabolism [19].

### 4.1. Renal Tubular Epithelial Cells and Proteinuria

The tubular epithelium contains some of the most energy-demanding cells in the body because they perform massive tubular reabsorption, and thus they critically depend on FAO [20]. When saturated FA (SFA) such as PA or stearic acid is completely oxidized to CO2 and water, more molecules of ATP are produced per carbon atom than by the similar oxidation of glucose. Thus, high-energy demanding tissues, such as cardiac and skeletal muscle, preferentially take up and oxidize FA as an energy source. 

Epidemiological studies have identified urinary albumin as an aggravating factor in CKD [21]. In the context of albuminuria toxicity, the low-molecular-weight protein itself may damage tubulointerstitial compartments. However, given that serum FAs are bound to serum albumin and DL occasionally accompanies proteinuric kidney diseases, urinary albumin-bound FA overload may also cause tubulointerstitial damage, according to the lipid nephrotoxicity hypothesis proposed in 1982 [22]. Accumulating evidence indicates that intracellular lipid deposits cause proximal tubule cell dysfunction in proteinuric conditions [23]. Moreover, while infusion of albumin in animal models [24,25] results in apoptosis, cytotoxicity is attenuated by administration of delipidated albumin [26,27,28].

Any factors involved in increased uptake, increased synthesis, and diminished β-oxidation of FA may contribute to tubular lipotoxicity. Although most filtered albumin is reabsorbed by the brush border complex of megalin/cubilin/amnionless, a different transport machinery is thought to be associated with this cytotoxicity. In humans, the protein cluster of differentiation 36/FA translocase (CD36/FAT) mediates tubular cell apoptosis induced by glycated albumins (AGE-BSA and CML-BSA) and PA through sequential kinase activation [29]. Although CD36/FAT is not expressed in the mouse kidney proximal tubules, transgenic expression of CD36/FTA in proximal tubular cells led to considerable accumulation of intracellular lipids, including TAG [30]. Generally, FAs are internalized by vectorial acylation, which involves simultaneous transport (likely by a member of the FA transporter family) and esterification to long chain fatty acyl-CoA (LCA-CoA) by plasma-membrane LCA-CoA synthetases. In the kidney tubules, the simultaneous uptake and metabolism of FA are mediated by LCF-CoA ligase 1, encoded by ACSL1, and FATP2, encoded by SLC27A2. Silencing of SLC27A2 in human kidney proximal tubular epithelial cells led to increased Oil Red O staining and subsequent apoptosis following when exposure to FA. Further, tubular epithelial cell apoptosis in lipidated albumin–injected mice was reduced in Slc27a2-deficient mice [31].

As described earlier in Figure 1, LCA-CoA is transported by carnitine shuttle into mitochondrial matrix for FAO. Although it primarily produces large amounts of ATP, mitochondrial FAO is also the source of the increased net reactive oxtgen species (ROS) production causing early renal damage with diabetes [32,33], Also in cases in which the ATP demand is met, LCA-CoA converts diacylglycerol (DAG) to TAG, which is stored in intracellular lipid droplets. If the CPT-dependent mitochondrial pathway and conversion to DAG or TAG are blocked or saturated, accumulated LCA-CoAs become toxic and lead to tubular cell apoptosis, known as lipoapoptosis. Notably, disruption of the Na^+^/H^+^ exchanger 1 (NHE1)-phosphatidylinositol 4,5-bisphosphate (PIP2) interaction-mediated cell survival and ceramide formation has been suggested as a mechanism [27]. 

Technically, as a result of kidney dysfunction, enhancement of tubular FA synthesis elicits lipid accumulation. Sterol regulatory element-binding protein (SREBP)-1c [34] and carbohydrate response element-binding protein (ChREBP) transcription factors [34,35] contribute to increase TAG content in cultured tubular cells. Although increased expression of these enzymes in the diabetic kidney remains controversial, accelerated lipogenesis should be investigated further as a cause of tubular lipotoxicity.

Pharmacological and genetic studies have suggested that stimulation to enhance FA utilization can improve histopathology and slow CKD progression. Thus, transcriptome studies of diseased fibrotic kidney samples highlighted low FA and carbohydrate metabolism and high intracellular lipid accumulation. Tubular transgenic expression of peroxisome proliferator-activated receptor gamma coactivator 1-alpha (Ppargc1a) normalized expression of CPT, presumably promoting FAO. Nevertheless, fenofibrate treatment of mice with fibrotic kidneys substantially upregulated the expression of the CPT genes Cpt1a and Cpt2, as well as the expression of Acox1 and Acox2, which mediated FAO, reduced fibrosis, and improved renal function, while the levels of enzymes involved in glucose metabolism were unchanged [30].

Additionally, the kidney exhibits the highest fractional rate of protein synthesis of any organ in the body. Thus, the endoplasmic reticulum (ER) of renal cells is subjected to greater stresses than the ER of cells in other organs. ER dysfunction leads to a multifactorial type of kidney damage, known as ER stress [36,37,38,39]. Our group demonstrated that albumin stimuli deteriorated renal tubular cell viability and enhanced the expression of ER stress-related glucose-regulated protein 78 (GRP78) and oxygen-regulated protein 150 (ORP150) mRNAs and interestingly increased apoptosis and activation of caspase-12 [36]. Uremic toxin also induced ER stress in cultured human proximal tubular cells, demonstrated by the increase in C/EBP homologous protein (CHOP) [37]. More importantly [40], PA causes both oxidative and ER stress characterized by CHOP expression in cultured renal tubular epithelial cells, leading to lipoapoptosis [41]. Thus, exposure of tubule cells to severe or long-term stress induces unfolded protein response (UPR) pathway-mediated apoptosis and leads to CKD progression [36,37,42,43].

Exploitation of activation of the unfolded protein response (UPR) pathway is another promising intervention for downregulating mitochondrial FAO [40]. Activating transcription factor (ATF) 6α, a transcription factor that functions in one of the three arms of the UPR pathway, enhances the transcriptional activity of peroxisome proliferator-activated receptor α (PPARα) and triggers activation of PPARα downstream targets, including CPT1α and MCAD, in hepatocytes [44]. We recently showed that pathogenic ATF6α activation downregulated mitochondrial FAO activity by suppressing PPARα in renal tubule cells and subsequent intracellular lipid accumulation in peritubular capillaries [40].

### 4.2. Renal Interstitial Cells and Anemia

The renal interstitium is defined as the space excluding the glomeruli, tubules, and capillaries in the kidney. It is bounded on all sides by tubular and vascular basement membranes and filled with cells, extracellular matrix, and interstitial fluid [45]. The intertubular interstitium is composed of multiple cell types including fibroblasts, macrophages, dendritic cells, lymphocytes, and lymphatic endothelial cells [46]. Compared to the kidney tubular epithelium, lipotoxicity in the kidney interstitium is not well-understood.

The kidney was long regarded as a primary source of erythropoietin (EPO) and renal anemia is frequently accompanied by CKD as mentioned above [47]. After basic research long struggled to evaluate the very low EPO expression in the organ, transgenic animals expressing an EPO transgene fused to a green fluorescent protein reporter tag enabled visualization of renal interstitial fibroblasts as a source of EPO [48,49,50].

Our group demonstrated the effect of PA on EPO production and ER stress pathway [51]. Intriguingly, EPO production was suppressed by PA-conjugated bovine serum albumin (BSA) or a high-PA diet, but not by oleic acid (OA)-conjugated BSA or a high-OA diet [51]. Silencing of ATF4, a key regulator of autophagy in response to amino acid starvation or ER stress [52], reversed the suppressive effect of PA on EPO production. Transgenic super-anemic mice (ISAM) mated with EPO-Cre mice to provide lineage labeling of renal EPO producing (REP) cells which clarified that ATF4 activation by PA reduced REP cell EPO production and release. Thus, ER stress induced by PA suppressed EPO expression by REP cells, highlighting the importance of the links between ER stress, DL, and hypoxia in the development and progression of anemia in CKD.

### 4.3. Renal Glomerular Podocytes: Perspectives in Diabetes

Podocytes are visceral glomerular epithelial cells that play an essential role in maintaining the glomerular tuft and filtration barrier. The most significant evidence for this role came from studying the phenotypes of mutations in genes exclusively expressed in podocytes within the kidney, which disrupted the integrity of the glomerular filter. In addition to these congenital disorders of podocytes, it is interesting that acquired obesity leads to glomerulopathy with glomerular hypertrophy, mesangial matrix expansion, and focal segmental glomerulosclerosis. Proposed mechanisms leading to renal pathology include abnormal lipid metabolism, lipotoxicity, inhibition of AMP kinase, and ER stress [53,54]. Furthermore, mice fed with a high-fat diet (HFD) showed dramatic changes in their podocyte mitochondrial structure, which were reversed by a tetrapeptide that targets cardiolipin and protects the mitochondrial cristae structure [55]. This finding indicates that mitochondrial damage is a product of lipotoxicity. Several interventional approaches have been suggested to mitigate podocyte lipotoxicity in DN. Falkevall et al. found that renal vascular endothelial growth factor-B (VEGF-B) expression is correlated with the severity of disease in experimental mouse models of DKD and that blocking VEGF-B signaling slowed the development of DKD-associated pathologies in mice [56].

A longitudinal cohort study revealed that the blood levels of the tumor necrosis factor (TNF) receptors TNFR1 and TNFR2 predicted the long-term progression of DKD, although how increased TNF promotes injury remains unclear [57,58]. Recently, Pedigo et al. found that TNF promotes free cholesterol–dependent podocyte apoptosis via a nuclear factor of activated T cells 1(NFATc1)/ATP-binding cassette protein A1 (ABCA1)-dependent mechanism. Interestingly, podocyte-specific ABCA1 deficiency in vivo decreased TNF-induced albuminuria, which was partially prevented by cholesterol depletion. Thus, their findings suggest that agents targeting cholesterol are promising for reducing proteinuria [59].

ROS are important mediators of lipid-induced podocyte injury. PA induces mitochondrial superoxide and hydrogen peroxide (H_2_O_2_) generation in cultured podocytes, while OA inhibits PA-induced ROS formation [60]. Long-term exposure of podocytes to PA reduced the protein levels of paired-related homeobox genes, glutathione peroxidase, and catalase, which are all capable of scavenging ROS. In the glomeruli of patients with advanced diabetes, H_2_O_2_ generation was elevated and the expression of these proteins was enhanced. Transforming growth factor-β1 (TGF-β1) in podocytes may also promote mesangial matrix expansion, suggesting that podocytes are susceptible to PA-induced oxidative damage with impaired peroxidase activity and that peroxidases have negligible antioxidant effects in podocytes during the late stages of DN [61].

In addition, adiponectin, a humoral factor released from the adipose tissue, may play a pathogenic role in lipotoxicity-induced podocyte injury as the receptor agonist can inhibit renal cell apoptosis in *db*/*db* mice [62]. In high glucose-treated murine podocytes, adiponectin increases intracellular Ca^2+^ concentration, which in turn activates a CaMKKβ/phosphorylated Ser431LKB1/phosphorylated Thr172AMPK/PPARα pathway and downstream signaling, decreasing high glucose-induced oxidative stress [63].

## 5. Lipotoxicity in Cardiomyocytes

A high incidence of cardiovascular morbidity and mortality is common among patients with CKD [64], which is largely explained by accelerated cardiac hypertrophic changes [65] and atherosclerosis [66] associated with renal dysfunction and impaired homeostasis. Given that the DL incidence is very high in patients with ESKD [67,68], it is reasonable to speculate that lipotoxicity bridges CKD and cardiovascular pathology.

The heart requires substantial energy to continuously pump against a systemic afterload. In addition to the ATP converted to mechanical energy by myosin for pumping, the heart also consumes a significant amount of ATP to maintain cardiomyocyte membrane potential and to drive the reuptake of Ca^2+^ via the sarco/endoplasmic reticulum Ca^2+^-ATPase (SERCA). To meet this energy demand, the heart is well-supplied with oxygen and nutrients, with more than 90% of ATP production dependent on aerobatic oxidation of mitochondrial substrates. In the healthy adult heart, 60–80% of ATP production is estimated to depend on FAO [69]. FA secreted from visceral fat passes through the cardiovascular system, indicating that FAs contribute to the activation of inflammation in cardiovascular and metabolic tissues. FA is taken up by cardiac myocytes through CD36 and the FA binding protein (FABP), and then bound to FABP or converted to acyl-CoA. Most FA in the cell is utilized as a substrate for mitochondrial oxidation, while some portion of FA is converted to TAG and accumulates in the intracellular lipid droplets [70].

Given that myocardial TAG accumulation leads to deteriorated cardiac function, intracellular TAG accumulation manifests lipotoxicity [71]. In this context, ER stress negatively influences cardiomyocytes upon stress-mediated lipid accumulation in response to harsh conditions, including pressure overload [72] and ischemia [73]. This hypothesis is supported by the demonstration that very low density lipoprotein receptor (VLDLR) depletion reduces the accumulation of ischemia-induced lipids in mouse hearts [74]. These results suggest that VLDLR-induced lipid accumulation in the ischemic heart causes cell death by activating ER stress. Drevinge et al. showed increased intracellular cholesteryl ester levels, ER stress, UPR signaling activation, and cardiac dysfunction in a pig model with cardiac ischemia [75]. In addition to ischemic injury, SFAs induce ER stress and apoptosis in cardiomyocytes. Cell culture experiments demonstrated that PA treatment causes ER stress [76], which was prevented by agonist activation of PPARβ/δ. PA-induced, ER stress-mediated lipotoxicity in cardiomyocytes occurred by inhibiting the sequestration of FA in lipid droplets [77,78]. Additionally, *Pparβ*/*δ* knockout mice fed an HFD displayed ER stress, suggesting a protective role of PPARβ/δ in ER stress-mediated lipotoxicity. These results suggest that ER stress can explain lipotoxicity in CHF or HF. Fat accumulation in the heart decreased the expression of PPARα [79]. In rats with obesity resulting from a loss-of-function mutation in the gene encoding for the leptin receptor (Zucker Diabetic Fatty, genotype *fa*/*fa*), myocardial TAG was elevated by reduced expression of FAO enzymes and PPARα, together with apoptosis markers. Interestingly, these changes were reversed by troglitazone therapy. Recently, Tsushima et al. established a transgenic mouse model of cardiac lipotoxicity by overexpressing *ACSL1* in cardiomyocytes. They analyzed the mitochondrial morphology and metabolism, showing that an increase in myocardial FA uptake reduced the minimum mitochondrial diameters, which was associated with increased palmitoyl-carnitine oxidation and increased ROS generation. While initially activating mitochondrial respiration and increasing both mitochondrial polarization and ATP synthesis, PA exposure of neonatal rat ventricular cardiomyocytes for >8 h enhanced ROS generation, which was accompanied by loss of the mitochondrial reticulum and a pattern suggesting increased mitochondrial fission. These data indicate that lipid overload-induced mitochondrial ROS generation causes mitochondrial dysfunction by inducing posttranslational modifications of mitochondrial proteins that regulate mitochondrial dynamics [80].

While PPARβ/δ and PPARα have been well analyzed in the context of cardiac lipotoxicity, PPARγ controls adipose tissue expansion and lipotoxicity in the periphery [81]. Genetic deletion of PPARγ2 in the *ob*/*ob* mice reduced fat mass while worsening insulin resistance and DL. This result indicates that a PPARγ2 agonist may prevent lipotoxicity. Intriguingly, doxorubicin inhibited PPARγ in transgenic diabetic mice [82], suggesting that doxorubicin-induced cardiac toxicity can be partly explained by lipotoxicity.

## 6. Lipotoxicity in Skeletal Muscle: Perspectives in Diabetes and Uremic Sarcopenia

The skeletal muscle is a pivotal endocrine organ as well as an essential component to produce a physical power. Skeletal muscle is responsible for approximately 80% of postprandial insulin-stimulated glucose disposal [83] and is an important organ in FA consumption associated with mitochondrial β-oxidation [84]. As a consequence of absorbed excess dietary FA and increased release of FA from dysfunctional adipose tissue, lipid droplets located within myocytes are not only classified as intramyocellular lipid (IMCL), chiefly in the form of TAG, but also as lipid intermediates such as LC-CoA, DAG, sphingolipids, phospholipids, and ceramides [85,86]. Oversupply of FA and a reduced rate of β-oxidation induce accumulation of IMCL [87], resulting in insulin resistance, inflammation, and apoptosis in skeletal muscle cells [88,89,90].

DN or DKD are the most common underlying diseases of CKD. Diabetes has an elevated accumulation of IMCL relative to lean individuals, and other reports have shown negative correlations [91,92]. Both experimental and clinical studies suggested that accumulation of DAG [93,94,95] and ceramides [96,97] are also negatively correlated with insulin sensitivity, but these results are controversial [98,99]. Hoeks et al. revealed that intravenous MCFA administration to healthy human subjects does not lead to increased ceramide and IMCL levels, but promotes insulin resistance [98]. By contrast, high-performance liquid chromatography-tandem mass spectrometry of human skeletal muscular tissues performed by Coen et al. showed that skeletal muscle insulin resistance was associated with greater IMTG content in type I but not type II fibers [97].

Moreover, the skeletal muscle of trained athletes, despite its elevated lipid content, is significantly insulin-sensitive with a high oxidative capacity, a phenomenon known as the athlete’s paradox [100]. During prolonged, moderate-intensity exercise in trained human subjects, 60–70% of intramuscular TAG was depleted in type I fibers (slow-twitch and rich in mitochondria), accounting for up to 50% of total lipid oxidized as a fuel source in the muscle [101]. Furthermore, the IMCL content is negatively correlated with insulin sensitivity only in type I fibers. Therefore, the total quantity of IMCL may not be the critical parameter for insulin resistance, but rather these effects are dictated by the locations of lipid pools and oxidative capacity.

Excess FA induces an increase in mitochondrial β-oxidation, leading to incomplete FAO and increased ROS. In contrast, increased ROS levels in skeletal muscle plus exercise activate redox-sensitive kinases including AMP-activated protein kinase (AMPK) [102]. AMPK activity elevates levels of the peroxisome proliferator-activated receptor-γ coactivator-1α (PGC-1α), a key regulator of mitochondrial biogenesis [103,104]. Increasing the mitochondrial content of cultured cells reduces their oxygen consumption [105]. PGC-1α is also a principal factor in the determination of muscle-fiber type, changing the distribution to favor type I fibers [106], leading to improved exercise capacity via increased FA utilization [107]. Furthermore, impairing FAO by depleting CPT1 in skeletal muscle leads to activation of AMPK and PGC1α. This in turn induces adaptive metabolic responses in the skeletal muscle with increased mitochondrial biogenesis, oxidative capacity, compensatory peroxisomal fat oxidation, and amino acid catabolism [108]. Despite an elevated plasma lipid level and accumulation of both IMCL and lipotoxic species, fasting insulin and glucose levels are lower when glucose utilization is enhanced. Although additional studies are needed to determine the detailed mechanisms, activation of AMPK and PGC-1α and regulation of CPT1 may be very important in preventing lipotoxicity.

It remains controversial whether ER stress plays a pivotal role in lipotoxicity in the skeletal myocyte. Laboratory experimental analyses, including some using human samples, suggest that ER stress has little association with lipid-induced skeletal myocyte dysfunction [109,110,111,112]. In contrast, cultured cell experiments revealed that PA treatment induced ER stress, which was reversed by stearoyl-CoA desaturase 1 (SCD1) expression [113]. In mice, HFD intake upregulated skeletal BiP and ATF4/CHOP expression [114]. Intriguingly, ER stress relievers such as tauroursodeoxycholic acid (TUDCA) [109] and 4-phenylbutyric acid (PBA) [115] increased muscle insulin sensitivity in patients with obesity, supporting the hypothesis of ER stress-mediated lipotoxicity in the skeletal muscle.

A decline in the skeletal muscle mass, strength, and endurance is known as sarcopenia, which has been well studied in the context of aging. Today, the clinical significance of sarcopenia is highlighted in patients with CKD, even those not on dialysis [116]. Epidemiological studies revealed a high incidence of sarcopenia in elderly hemodialysis (HD) patients [117], with the incidence of sarcopenia increasing as renal function deteriorates [118]. The importance of uremic sarcopenia lies in its impact on mortality and morbidity, also affecting the quality of life for patients with CKD [119], cardiovascular events [120], and overall survival [121]. The etiology may be explained by the catalytic state associated with protein wasting and with multiple metabolic derangements. Uremic toxins such as indoxyl sulfate and p-cresol, accumulate as renal clearance deteriorates, which has been proposed to have toxic effects on skeletal muscle function [122,123,124,125]. Form the viewpoint of lipotoxicity, it remains unclear which factors contribute to muscle mass reduction in uremic status, while multifaceted aspects of lipotoxicity have been extensively studied in skeletal muscle. First, abnormal lipid metabolism and obesity are frequently associated with sarcopenia in patients with advanced CKD [126]. Yuan et al. found that accumulation of truncal fat mass was associated with elevated levels of circulating hepatic growth factor (HGF), a marker for obesity in the general population, and in the presence of protein-energy wasting predicted higher mortality [127]. Other underlying mechanisms remain unknown, although some phenomena are partially explained by altered mineral metabolism, sustained inflammation, elevated oxidative/nitrative stress [128], malnutrition, and accumulated uremic toxins [122,123,124,129,130]. Recently, Jheng et al. focused on ER stress in this context and clarified that uremic toxin not only promotes expression of E3 ubiquitin ligases, but also stimulates eIF2α phosphorylation and XBP1 mRNA splicing in the UPR [125]. This phenomenon appears reasonable as a cause, as sarcopenia is caused by an imbalance of skeletal protein synthesis and degradation.

Whether the modulation of lipid metabolism is beneficial in the treatment of uremic sarcopenia is worthy of investigation. Ghrelin, a hormone produced by the gastrointestinal tract, regulates food intake and energy homeostasis. It is the only known peptide hormone modified by a fatty acid. In the gastrocnemius muscle in 5/6 nephrectomized rats, Gortan Cappellari et al. showed that treatment with unacylated ghrelin enhanced mitophagy, normalized oxidative stress, and reduced inflammation, and impaired insulin signaling as well as muscle loss [131]. In addition, a recent clinical study with CKD patients demonstrated that a higher ratio of n-6/n-3 polyunsaturated fatty acids (PUFAs), characteristic of the Western diet, in CKD patients is an independent risk factor for reduced appendicular skeletal muscle mass, suggesting that increasing quantities of n-3 PUFAs in the diet can help normalize the ratio [132]

## 7. Conclusions

Patients with CKD even before ESKD have abnormal lipid profiles, which are risk factors for atherosclerosis and cardiovascular events and promote ectopic lipid deposits and dysregulated handling of lipid molecules in multiple end-organs including the kidney, heart, and skeletal muscle. Accumulated lipids in non-adipose tissue in the uremic milieu disrupt homeostasis.

This review is unique in that lipotoxicity is discussed in the heart and skeletal muscle as well as in the kidney in uremic conditions, while most reviews of lipotoxicity focus on the adipose tissue, pancreas, and liver, particularly in terms of obesity and diabetes. Thus, the current review is limited by the insufficient discussion of research publications highlighting CKD and lipotoxicity.

Attenuation of lipotoxicity by eradicating ectopic lipid deposits may contribute to self-tissue protection. Current therapeutics treating DL, including dietary changes and administration of lipid-lowering drugs, may need to be reevaluated in this regard. Additionally, basic research findings indicate that ER stress and the UPR pathways underlie lipotoxicity in peripheral tissues, which could be a future point of therapeutic intervention. Nevertheless, as the significance of lipid lowering therapy to improve survival remains controversial, particularly in ESKD [133], studies with greater clinical relevance are needed. Further studies of lipotoxicity mechanisms may yield approaches to reduce tissue damage.

## References

[1] Ludwig D.S. (2002). The glycemic index: Physiological mechanisms relating to obesity, diabetes, and cardiovascular disease. JAMA.

[2] Schwartz M.W., Seeley R.J., Zeltser L.M., Drewnowski A., Ravussin E., Redman L.M., Leibel R.L. (2017). Obesity Pathogenesis: An Endocrine Society Scientific Statement. Endocr. Rev..

[3] Bosy-Westphal A., Hagele F., Nas A. (2017). Impact of dietary glycemic challenge on fuel partitioning. Eur. J. Clin. Nutr..

[4] Ebbeling C.B., Feldman H.A., Klein G.L., Wong J.M.W., Bielak L., Steltz S.K., Luoto P.K., Wolfe R.R., Wong W.W., Ludwig D.S. (2018). Effects of a low carbohydrate diet on energy expenditure during weight loss maintenance: Randomized trial. BMJ.

[5] Ludwig D.S., Willett W.C., Volek J.S., Neuhouser M.L. (2018). Dietary fat: From foe to friend?. Science.

[6] Tovar A.R., Torres N. (2010). The role of dietary protein on lipotoxicity. Biochim. Biophys. Acta.

[7] Sirtori C.R., Galli C., Anderson J.W., Arnoldi A. (2009). Nutritional and nutraceutical approaches to dyslipidemia and atherosclerosis prevention: Focus on dietary proteins. Atherosclerosis.

[8] Inoue N., Nagao K., Sakata K., Yamano N., Gunawardena P.E., Han S.Y., Matsui T., Nakamori T., Furuta H., Takamatsu K. (2011). Screening of soy protein-derived hypotriglyceridemic di-peptides in vitro and in vivo. Lipids Health Dis..

[9] Parafati M., Lascala A., Morittu V.M., Trimboli F., Rizzuto A., Brunelli E., Coscarelli F., Costa N., Britti D., Ehrlich J. (2015). Bergamot polyphenol fraction prevents nonalcoholic fatty liver disease via stimulation of lipophagy in cafeteria diet-induced rat model of metabolic syndrome. J. Nutr. Biochem..

[10] Parafati M., Lascala A., La Russa D., Mignogna C., Trimboli F., Morittu V.M., Riillo C., Macirella R., Mollace V., Brunelli E. (2018). Bergamot Polyphenols Boost Therapeutic Effects of the Diet on Non-Alcoholic Steatohepatitis (NASH) Induced by "Junk Food": Evidence for Anti-Inflammatory Activity. Nutrients.

[11] Musso G., Cassader M., Paschetta E., Gambino R. (2018). Bioactive Lipid Species and Metabolic Pathways in Progression and Resolution of Nonalcoholic Steatohepatitis. Gastroenterology.

[12] Garbarino J., Sturley S.L. (2009). Saturated with fat: New perspectives on lipotoxicity. Curr. Opin. Clin. Nutr. Metab. Care.

[13] Lim S., Meigs J.B. (2013). Ectopic fat and cardiometabolic and vascular risk. Int J. Cardiol..

[14] Tsimihodimos V., Dounousi E., Siamopoulos K.C. (2008). Dyslipidemia in chronic kidney disease: An approach to pathogenesis and treatment. Am. J. Nephrol..

[15] Bakris G.L. (2012). Lipid disorders in uremia and dialysis. Contrib. Nephrol..

[16] Unger R.H., Zhou Y.T. (2001). Lipotoxicity of beta-cells in obesity and in other causes of fatty acid spillover. Diabetes.

[17] Unger R.H., Clark G.O., Scherer P.E., Orci L. (2010). Lipid homeostasis, lipotoxicity and the metabolic syndrome. Biochim. Biophys. Acta.

[18] Kramer H.J., Saranathan A., Luke A., Durazo-Arvizu R.A., Guichan C., Hou S., Cooper R. (2006). Increasing body mass index and obesity in the incident ESRD population. J. Am. Soc. Nephrol..

[19] Upadhyay A., Earley A., Lamont J.L., Haynes S., Wanner C., Balk E.M. (2012). Lipid-lowering therapy in persons with chronic kidney disease: A systematic review and meta-analysis. Ann. Intern. Med..

[20] Meyer C., Nadkarni V., Stumvoll M., Gerich J. (1997). Human kidney free fatty acid and glucose uptake: Evidence for a renal glucose-fatty acid cycle. Am. J. Physiol..

[21] Risdon R.A., Sloper J.C., De Wardener H.E. (1968). Relationship between renal function and histological changes found in renal-biopsy specimens from patients with persistent glomerular nephritis. Lancet.

[22] Dupont B., Oberfield S.E., Smithwick E.M., Lee T.D., Levine L.S. (1977). Close genetic linkage between HLA and congenital adrenal hyperplasia (21-hydroxylase deficiency). Lancet.

[23] Weinberg J.M. (2006). Lipotoxicity. Kidney Int..

[24] Schelling J.R., Nkemere N., Kopp J.B., Cleveland R.P. (1998). Fas-dependent fratricidal apoptosis is a mechanism of tubular epithelial cell deletion in chronic renal failure. Lab. Investig..

[25] Van Timmeren M.M., Bakker S.J., Stegeman C.A., Gans R.O., van Goor H. (2005). Addition of oleic acid to delipidated bovine serum albumin aggravates renal damage in experimental protein-overload nephrosis. Nephrol. Dial. Transplant..

[26] Kamijo A., Kimura K., Sugaya T., Yamanouchi M., Hase H., Kaneko T., Hirata Y., Goto A., Fujita T., Omata M. (2002). Urinary free fatty acids bound to albumin aggravate tubulointerstitial damage. Kidney Int..

[27] Khan S., Abu Jawdeh B.G., Goel M., Schilling W.P., Parker M.D., Puchowicz M.A., Yadav S.P., Harris R.C., El-Meanawy A., Hoshi M. (2014). Lipotoxic disruption of NHE1 interaction with PI(4,5)P2 expedites proximal tubule apoptosis. J. Clin. Investig..

[28] Ruggiero C., Elks C.M., Kruger C., Cleland E., Addison K., Noland R.C., Stadler K. (2014). Albumin-bound fatty acids but not albumin itself alter redox balance in tubular epithelial cells and induce a peroxide-mediated redox-sensitive apoptosis. Am. J. Physiol. Renal Physiol..

[29] Susztak K., Ciccone E., McCue P., Sharma K., Bottinger E.P. (2005). Multiple metabolic hits converge on CD36 as novel mediator of tubular epithelial apoptosis in diabetic nephropathy. PLoS Med..

[30] Kang H.M., Ahn S.H., Choi P., Ko Y.A., Han S.H., Chinga F., Park A.S., Tao J., Sharma K., Pullman J. (2015). Defective fatty acid oxidation in renal tubular epithelial cells has a key role in kidney fibrosis development. Nat. Med..

[31] Khan S., Cabral P.D., Schilling W.P., Schmidt Z.W., Uddin A.N., Gingras A., Madhavan S.M., Garvin J.L., Schelling J.R. (2018). Kidney Proximal Tubule Lipoapoptosis Is Regulated by Fatty Acid Transporter-2 (FATP2). J. Am. Soc. Nephrol..

[32] Rosca M.G., Vazquez E.J., Chen Q., Kerner J., Kern T.S., Hoppel C.L. (2012). Oxidation of fatty acids is the source of increased mitochondrial reactive oxygen species production in kidney cortical tubules in early diabetes. Diabetes.

[33] La Russa D., Giordano F., Marrone A., Parafati M., Janda E., Pellegrino D. (2019). Oxidative Imbalance and Kidney Damage in Cafeteria Diet-Induced Rat Model of Metabolic Syndrome: Effect of Bergamot Polyphenolic Fraction. Antioxidants (Basel).

[34] Sun L., Halaihel N., Zhang W., Rogers T., Levi M. (2002). Role of sterol regulatory element-binding protein 1 in regulation of renal lipid metabolism and glomerulosclerosis in diabetes mellitus. J. Biol. Chem..

[35] Proctor G., Jiang T., Iwahashi M., Wang Z., Li J., Levi M. (2006). Regulation of renal fatty acid and cholesterol metabolism, inflammation, and fibrosis in Akita and OVE26 mice with type 1 diabetes. Diabetes.

[36] Ohse T., Inagi R., Tanaka T., Ota T., Miyata T., Kojima I., Ingelfinger J.R., Ogawa S., Fujita T., Nangaku M. (2006). Albumin induces endoplasmic reticulum stress and apoptosis in renal proximal tubular cells. Kidney Int..

[37] Kawakami T., Inagi R., Wada T., Tanaka T., Fujita T., Nangaku M. (2010). Indoxyl sulfate inhibits proliferation of human proximal tubular cells via endoplasmic reticulum stress. Am. J. Physiol. Renal Physiol..

[38] Maekawa H., Inagi R. (2017). Stress Signal Network between Hypoxia and ER Stress in Chronic Kidney Disease. Front. Physiol..

[39] Inoue T., Maekawa H., Inagi R. (2019). Organelle crosstalk in the kidney. Kidney Int..

[40] Jao T.M., Nangaku M., Wu C.H., Sugahara M., Saito H., Maekawa H., Ishimoto Y., Aoe M., Inoue T., Tanaka T. (2019). ATF6alpha downregulation of PPARalpha promotes lipotoxicity-induced tubulointerstitial fibrosis. Kidney Int..

[41] Katsoulieris E., Mabley J.G., Samai M., Sharpe M.A., Green I.C., Chatterjee P.K. (2010). Lipotoxicity in renal proximal tubular cells: Relationship between endoplasmic reticulum stress and oxidative stress pathways. Free Radic. Biol. Med..

[42] Lindenmeyer M.T., Rastaldi M.P., Ikehata M., Neusser M.A., Kretzler M., Cohen C.D., Schlondorff D. (2008). Proteinuria and hyperglycemia induce endoplasmic reticulum stress. J. Am. Soc. Nephrol..

[43] Ozkok A., Edelstein C.L. (2014). Pathophysiology of cisplatin-induced acute kidney injury. Biomed. Res. Int..

[44] Chen X., Zhang F., Gong Q., Cui A., Zhuo S., Hu Z., Han Y., Gao J., Sun Y., Liu Z. (2016). Hepatic ATF6 Increases Fatty Acid Oxidation to Attenuate Hepatic Steatosis in Mice Through Peroxisome Proliferator-Activated Receptor alpha. Diabetes.

[45] Lemley K.V., Kriz W. (1991). Anatomy of the renal interstitium. Kidney Int..

[46] Nelson P.J., Rees A.J., Griffin M.D., Hughes J., Kurts C., Duffield J. (2012). The renal mononuclear phagocytic system. J. Am. Soc. Nephrol..

[47] Zeisberg M., Kalluri R. (2015). Physiology of the Renal Interstitium. Clin. J. Am. Soc. Nephrol..

[48] Kurtz A., Eckardt K.U., Neumann R., Kaissling B., Le Hir M., Bauer C. (1989). Site of erythropoietin formation. Contrib. Nephrol..

[49] Obara N., Suzuki N., Kim K., Nagasawa T., Imagawa S., Yamamoto M. (2008). Repression via the GATA box is essential for tissue-specific erythropoietin gene expression. Blood.

[50] Pan X., Suzuki N., Hirano I., Yamazaki S., Minegishi N., Yamamoto M. (2011). Isolation and characterization of renal erythropoietin-producing cells from genetically produced anemia mice. PLoS ONE.

[51] Anusornvongchai T., Nangaku M., Jao T.M., Wu C.H., Ishimoto Y., Maekawa H., Tanaka T., Shimizu A., Yamamoto M., Suzuki N. (2018). Palmitate deranges erythropoietin production via transcription factor ATF4 activation of unfolded protein response. Kidney Int..

[52] B’Chir W., Maurin A.C., Carraro V., Averous J., Jousse C., Muranishi Y., Parry L., Stepien G., Fafournoux P., Bruhat A. (2013). The eIF2alpha/ATF4 pathway is essential for stress-induced autophagy gene expression. Nucleic Acids Res..

[53] Kim M.Y., Lim J.H., Youn H.H., Hong Y.A., Yang K.S., Park H.S., Chung S., Ko S.H., Shin S.J., Choi B.S. (2013). Resveratrol prevents renal lipotoxicity and inhibits mesangial cell glucotoxicity in a manner dependent on the AMPK-SIRT1-PGC1alpha axis in db/db mice. Diabetologia.

[54] Stern J.H., Rutkowski J.M., Scherer P.E. (2016). Adiponectin, Leptin, and Fatty Acids in the Maintenance of Metabolic Homeostasis through Adipose Tissue Crosstalk. Cell Metab..

[55] Szeto H.H., Liu S., Soong Y., Alam N., Prusky G.T., Seshan S.V. (2016). Protection of mitochondria prevents high-fat diet-induced glomerulopathy and proximal tubular injury. Kidney Int..

[56] Falkevall A., Mehlem A., Palombo I., Heller Sahlgren B., Ebarasi L., He L., Ytterberg A.J., Olauson H., Axelsson J., Sundelin B. (2017). Reducing VEGF-B Signaling Ameliorates Renal Lipotoxicity and Protects against Diabetic Kidney Disease. Cell Metab..

[57] Niewczas M.A., Gohda T., Skupien J., Smiles A.M., Walker W.H., Rosetti F., Cullere X., Eckfeldt J.H., Doria A., Mayadas T.N. (2012). Circulating TNF receptors 1 and 2 predict ESRD in type 2 diabetes. J. Am. Soc. Nephrol..

[58] Gohda T., Niewczas M.A., Ficociello L.H., Walker W.H., Skupien J., Rosetti F., Cullere X., Johnson A.C., Crabtree G., Smiles A.M. (2012). Circulating TNF receptors 1 and 2 predict stage 3 CKD in type 1 diabetes. J. Am. Soc. Nephrol..

[59] Pedigo C.E., Ducasa G.M., Leclercq F., Sloan A., Mitrofanova A., Hashmi T., Molina-David J., Ge M., Lassenius M.I., Forsblom C. (2016). Local TNF causes NFATc1-dependent cholesterol-mediated podocyte injury. J. Clin. Investig..

[60] Lee E., Choi J., Lee H.S. (2017). Palmitate induces mitochondrial superoxide generation and activates AMPK in podocytes. J. Cell Physiol..

[61] Lee E., Lee H.S. (2018). Peroxidase expression is decreased by palmitate in cultured podocytes but increased in podocytes of advanced diabetic nephropathy. J. Cell Physiol..

[62] Choi S.R., Lim J.H., Kim M.Y., Kim E.N., Kim Y., Choi B.S., Kim Y.S., Kim H.W., Lim K.M., Kim M.J. (2018). Adiponectin receptor agonist AdipoRon decreased ceramide, and lipotoxicity, and ameliorated diabetic nephropathy. Metabolism.

[63] Kim Y., Lim J.H., Kim M.Y., Kim E.N., Yoon H.E., Shin S.J., Choi B.S., Kim Y.S., Chang Y.S., Park C.W. (2018). The Adiponectin Receptor Agonist AdipoRon Ameliorates Diabetic Nephropathy in a Model of Type 2 Diabetes. J. Am. Soc. Nephrol..

[64] Hedayati S.S., Szczech L.A. (2004). The evaluation of underlying cardiovascular disease among patients with end-stage renal disease. Adv. Chronic Kidney Dis..

[65] Mimura I., Nishi H., Mise N., Mori M., Sugimoto T. (2010). Left ventricular geometry and cardiovascular mortality based on haemodialysis patient autopsy analyses. Nephrology (Carlton).

[66] Nakano T., Katsuki S., Chen M., Decano J.L., Halu A., Lee L.H., Pestana D.V.S., Kum A.S.T., Kuromoto R.K., Golden W.S. (2019). Uremic Toxin Indoxyl Sulfate Promotes Proinflammatory Macrophage Activation Via the Interplay of OATP2B1 and Dll4-Notch Signaling. Circulation.

[67] Iseki K., Yamazato M., Tozawa M., Takishita S. (2002). Hypocholesterolemia is a significant predictor of death in a cohort of chronic hemodialysis patients. Kidney Int..

[68] Russa D., Pellegrino D., Montesanto A., Gigliotti P., Perri A., Russa A., Bonofiglio R. (2019). Oxidative Balance and Inflammation in Hemodialysis Patients: Biomarkers of Cardiovascular Risk?. Oxid. Med. Cell Longev..

[69] Stanley W.C., Recchia F.A., Lopaschuk G.D. (2005). Myocardial substrate metabolism in the normal and failing heart. Physiol. Rev..

[70] Law B.A., Liao X., Moore K.S., Southard A., Roddy P., Ji R., Szulc Z., Bielawska A., Schulze P.C., Cowart L.A. (2018). Lipotoxic very-long-chain ceramides cause mitochondrial dysfunction, oxidative stress, and cell death in cardiomyocytes. FASEB J..

[71] Nielsen L.B., Perko M., Arendrup H., Andersen C.B. (2002). Microsomal triglyceride transfer protein gene expression and triglyceride accumulation in hypoxic human hearts. Arterioscler. Thromb Vasc. Biol.

[72] Okada K., Minamino T., Tsukamoto Y., Liao Y., Tsukamoto O., Takashima S., Hirata A., Fujita M., Nagamachi Y., Nakatani T. (2004). Prolonged endoplasmic reticulum stress in hypertrophic and failing heart after aortic constriction: Possible contribution of endoplasmic reticulum stress to cardiac myocyte apoptosis. Circulation.

[73] Azfer A., Niu J., Rogers L.M., Adamski F.M., Kolattukudy P.E. (2006). Activation of endoplasmic reticulum stress response during the development of ischemic heart disease. Am. J. Physiol. Heart Circ. Physiol..

[74] Perman J.C., Bostrom P., Lindbom M., Lidberg U., StAhlman M., Hagg D., Lindskog H., Scharin Tang M., Omerovic E., Mattsson Hulten L. (2011). The VLDL receptor promotes lipotoxicity and increases mortality in mice following an acute myocardial infarction. J. Clin. Investig..

[75] Drevinge C., Karlsson L.O., Stahlman M., Larsson T., Perman Sundelin J., Grip L., Andersson L., Boren J., Levin M.C. (2013). Cholesteryl esters accumulate in the heart in a porcine model of ischemia and reperfusion. PLoS ONE.

[76] Palomer X., Capdevila-Busquets E., Botteri G., Salvado L., Barroso E., Davidson M.M., Michalik L., Wahli W., Vazquez-Carrera M. (2014). PPARbeta/delta attenuates palmitate-induced endoplasmic reticulum stress and induces autophagic markers in human cardiac cells. Int. J. Cardiol..

[77] Bosma M., Dapito D.H., Drosatos-Tampakaki Z., Huiping-Son N., Huang L.S., Kersten S., Drosatos K., Goldberg I.J. (2014). Sequestration of fatty acids in triglycerides prevents endoplasmic reticulum stress in an in vitro model of cardiomyocyte lipotoxicity. Biochim. Biophys. Acta.

[78] Zou L., Li X., Wu N., Jia P., Liu C., Jia D. (2017). Palmitate induces myocardial lipotoxic injury via the endoplasmic reticulum stressmediated apoptosis pathway. Mol. Med. Rep..

[79] Zhou Y.T., Grayburn P., Karim A., Shimabukuro M., Higa M., Baetens D., Orci L., Unger R.H. (2000). Lipotoxic heart disease in obese rats: Implications for human obesity. Proc. Natl. Acad. Sci. USA.

[80] Tsushima K., Bugger H., Wende A.R., Soto J., Jenson G.A., Tor A.R., McGlauflin R., Kenny H.C., Zhang Y., Souvenir R. (2018). Mitochondrial Reactive Oxygen Species in Lipotoxic Hearts Induce Post-Translational Modifications of AKAP121, DRP1, and OPA1 That Promote Mitochondrial Fission. Circ. Res..

[81] Medina-Gomez G., Gray S.L., Yetukuri L., Shimomura K., Virtue S., Campbell M., Curtis R.K., Jimenez-Linan M., Blount M., Yeo G.S. (2007). PPAR gamma 2 prevents lipotoxicity by controlling adipose tissue expandability and peripheral lipid metabolism. PLoS Genet..

[82] Arunachalam S., Tirupathi Pichiah P.B., Achiraman S. (2013). Doxorubicin treatment inhibits PPARgamma and may induce lipotoxicity by mimicking a type 2 diabetes-like condition in rodent models. FEBS Lett..

[83] Baron A.D., Brechtel G., Wallace P., Edelman S.V. (1988). Rates and tissue sites of non-insulin- and insulin-mediated glucose uptake in humans. Am. J. Physiol..

[84] Watt M.J., Hoy A.J. (2012). Lipid metabolism in skeletal muscle: Generation of adaptive and maladaptive intracellular signals for cellular function. Am. J. Physiol. Endocrinol. Metab..

[85] Li Y., Xu S., Zhang X., Yi Z., Cichello S. (2015). Skeletal intramyocellular lipid metabolism and insulin resistance. Biophys. Rep..

[86] Ertunc M.E., Hotamisligil G.S. (2016). Lipid signaling and lipotoxicity in metaflammation: Indications for metabolic disease pathogenesis and treatment. J. Lipid. Res..

[87] Galgani J.E., Moro C., Ravussin E. (2008). Metabolic flexibility and insulin resistance. Am. J. Physiol. Endocrinol. Metab..

[88] Hulver M.W., Berggren J.R., Cortright R.N., Dudek R.W., Thompson R.P., Pories W.J., MacDonald K.G., Cline G.W., Shulman G.I., Dohm G.L. (2003). Skeletal muscle lipid metabolism with obesity. Am. J. Physiol. Endocrinol. Metab..

[89] Coll T., Eyre E., Rodriguez-Calvo R., Palomer X., Sanchez R.M., Merlos M., Laguna J.C., Vazquez-Carrera M. (2008). Oleate reverses palmitate-induced insulin resistance and inflammation in skeletal muscle cells. J. Biol. Chem..

[90] Turpin S.M., Lancaster G.I., Darby I., Febbraio M.A., Watt M.J. (2006). Apoptosis in skeletal muscle myotubes is induced by ceramides and is positively related to insulin resistance. Am. J. Physiol. Endocrinol. Metab..

[91] Eriksson J.W., Smith U., Waagstein F., Wysocki M., Jansson P.A. (1999). Glucose turnover and adipose tissue lipolysis are insulin-resistant in healthy relatives of type 2 diabetes patients: Is cellular insulin resistance a secondary phenomenon?. Diabetes.

[92] Spalding K.L., Arner E., Westermark P.O., Bernard S., Buchholz B.A., Bergmann O., Blomqvist L., Hoffstedt J., Naslund E., Britton T. (2008). Dynamics of fat cell turnover in humans. Nature.

[93] Itani S.I., Ruderman N.B., Schmieder F., Boden G. (2002). Lipid-induced insulin resistance in human muscle is associated with changes in diacylglycerol, protein kinase C, and IkappaB-alpha. Diabetes.

[94] Bergman B.C., Hunerdosse D.M., Kerege A., Playdon M.C., Perreault L. (2012). Localisation and composition of skeletal muscle diacylglycerol predicts insulin resistance in humans. Diabetologia.

[95] Moro C., Galgani J.E., Luu L., Pasarica M., Mairal A., Bajpeyi S., Schmitz G., Langin D., Liebisch G., Smith S.R. (2009). Influence of gender, obesity, and muscle lipase activity on intramyocellular lipids in sedentary individuals. J. Clin. Endocrinol. Metab..

[96] Straczkowski M., Kowalska I., Baranowski M., Nikolajuk A., Otziomek E., Zabielski P., Adamska A., Blachnio A., Gorski J., Gorska M. (2007). Increased skeletal muscle ceramide level in men at risk of developing type 2 diabetes. Diabetologia.

[97] Coen P.M., Dube J.J., Amati F., Stefanovic-Racic M., Ferrell R.E., Toledo F.G., Goodpaster B.H. (2010). Insulin resistance is associated with higher intramyocellular triglycerides in type I but not type II myocytes concomitant with higher ceramide content. Diabetes.

[98] Hoeks J., Mensink M., Hesselink M.K., Ekroos K., Schrauwen P. (2012). Long- and medium-chain fatty acids induce insulin resistance to a similar extent in humans despite marked differences in muscle fat accumulation. J. Clin. Endocrinol. Metab..

[99] Sogaard D., Ostergard T., Blachnio-Zabielska A.U., Baranowski M., Vigelso A.H., Andersen J.L., Dela F., Helge J.W. (2016). Training Does Not Alter Muscle Ceramide and Diacylglycerol in Offsprings of Type 2 Diabetic Patients Despite Improved Insulin Sensitivity. J. Diabetes Res..

[100] Goodpaster B.H., He J., Watkins S., Kelley D.E. (2001). Skeletal muscle lipid content and insulin resistance: Evidence for a paradox in endurance-trained athletes. J. Clin. Endocrinol. Metab..

[101] Van Loon L.J. (2004). Use of intramuscular triacylglycerol as a substrate source during exercise in humans. J. Appl. Physiol. (1985).

[102] Irrcher I., Ljubicic V., Hood D.A. (2009). Interactions between ROS and AMP kinase activity in the regulation of PGC-1alpha transcription in skeletal muscle cells. Am. J. Physiol. Cell Physiol..

[103] Wright D.C. (2007). Mechanisms of calcium-induced mitochondrial biogenesis and GLUT4 synthesis. Appl. Physiol. Nutr. Metab..

[104] Jager S., Handschin C., St-Pierre J., Spiegelman B.M. (2007). AMP-activated protein kinase (AMPK) action in skeletal muscle via direct phosphorylation of PGC-1alpha. Proc. Natl. Acad. Sci. USA.

[105] Lopez-Lluch G., Hunt N., Jones B., Zhu M., Jamieson H., Hilmer S., Cascajo M.V., Allard J., Ingram D.K., Navas P. (2006). Calorie restriction induces mitochondrial biogenesis and bioenergetic efficiency. Proc. Natl. Acad. Sci. USA.

[106] Li S., Lu A., Jia H. (2002). Therapeutic actions of the Chinese herbal formulae with cold and heat properties and their effects on ultrastructures of synoviocytes in rats of the collagen-induced arthritis. J. Tradit. Chin. Med..

[107] Tadaishi M., Miura S., Kai Y., Kano Y., Oishi Y., Ezaki O. (2011). Skeletal muscle-specific expression of PGC-1alpha-b, an exercise-responsive isoform, increases exercise capacity and peak oxygen uptake. PLoS ONE.

[108] Wicks S.E., Vandanmagsar B., Haynie K.R., Fuller S.E., Warfel J.D., Stephens J.M., Wang M., Han X., Zhang J., Noland R.C. (2015). Impaired mitochondrial fat oxidation induces adaptive remodeling of muscle metabolism. Proc. Natl. Acad. Sci. USA.

[109] Kars M., Yang L., Gregor M.F., Mohammed B.S., Pietka T.A., Finck B.N., Patterson B.W., Horton J.D., Mittendorfer B., Hotamisligil G.S. (2010). Tauroursodeoxycholic Acid may improve liver and muscle but not adipose tissue insulin sensitivity in obese men and women. Diabetes.

[110] Deldicque L., Van Proeyen K., Francaux M., Hespel P. (2011). The unfolded protein response in human skeletal muscle is not involved in the onset of glucose tolerance impairment induced by a fat-rich diet. Eur. J. Appl. Physiol..

[111] Hage Hassan R., Hainault I., Vilquin J.T., Samama C., Lasnier F., Ferre P., Foufelle F., Hajduch E. (2012). Endoplasmic reticulum stress does not mediate palmitate-induced insulin resistance in mouse and human muscle cells. Diabetologia.

[112] Rieusset J., Chauvin M.A., Durand A., Bravard A., Laugerette F., Michalski M.C., Vidal H. (2012). Reduction of endoplasmic reticulum stress using chemical chaperones or Grp78 overexpression does not protect muscle cells from palmitate-induced insulin resistance. Biochem. Biophys. Res. Commun..

[113] Peter A., Weigert C., Staiger H., Machicao F., Schick F., Machann J., Stefan N., Thamer C., Haring H.U., Schleicher E. (2009). Individual stearoyl-coa desaturase 1 expression modulates endoplasmic reticulum stress and inflammation in human myotubes and is associated with skeletal muscle lipid storage and insulin sensitivity in vivo. Diabetes.

[114] Deldicque L., Cani P.D., Philp A., Raymackers J.M., Meakin P.J., Ashford M.L., Delzenne N.M., Francaux M., Baar K. (2010). The unfolded protein response is activated in skeletal muscle by high-fat feeding: Potential role in the downregulation of protein synthesis. Am. J. Physiol. Endocrinol. Metab..

[115] Zhang H., Wang Y., Li J., Yu J., Pu J., Li L., Zhang H., Zhang S., Peng G., Yang F. (2011). Proteome of skeletal muscle lipid droplet reveals association with mitochondria and apolipoprotein a-I. J. Proteome. Res..

[116] Moorthi R.N., Avin K.G. (2017). Clinical relevance of sarcopenia in chronic kidney disease. Curr. Opin. Nephrol. Hypertens..

[117] Kim J.K., Choi S.R., Choi M.J., Kim S.G., Lee Y.K., Noh J.W., Kim H.J., Song Y.R. (2014). Prevalence of and factors associated with sarcopenia in elderly patients with end-stage renal disease. Clin. Nutr..

[118] Sharma D., Hawkins M., Abramowitz M.K. (2014). Association of sarcopenia with eGFR and misclassification of obesity in adults with CKD in the United States. Clin. J. Am. Soc. Nephrol..

[119] Morishita Y., Kubo K., Miki A., Ishibashi K., Kusano E., Nagata D. (2014). Positive association of vigorous and moderate physical activity volumes with skeletal muscle mass but not bone density or metabolism markers in hemodialysis patients. Int. Urol. Nephrol..

[120] Hanatani S., Izumiya Y., Onoue Y., Tanaka T., Yamamoto M., Ishida T., Yamamura S., Kimura Y., Araki S., Arima Y. (2018). Non-invasive testing for sarcopenia predicts future cardiovascular events in patients with chronic kidney disease. Int. J. Cardiol..

[121] Huang C.X., Tighiouart H., Beddhu S., Cheung A.K., Dwyer J.T., Eknoyan G., Beck G.J., Levey A.S., Sarnak M.J. (2010). Both low muscle mass and low fat are associated with higher all-cause mortality in hemodialysis patients. Kidney Int..

[122] Enoki Y., Watanabe H., Arake R., Sugimoto R., Imafuku T., Tominaga Y., Ishima Y., Kotani S., Nakajima M., Tanaka M. (2016). Indoxyl sulfate potentiates skeletal muscle atrophy by inducing the oxidative stress-mediated expression of myostatin and atrogin-1. Sci. Rep..

[123] Sato E., Mori T., Mishima E., Suzuki A., Sugawara S., Kurasawa N., Saigusa D., Miura D., Morikawa-Ichinose T., Saito R. (2016). Metabolic alterations by indoxyl sulfate in skeletal muscle induce uremic sarcopenia in chronic kidney disease. Sci. Rep..

[124] Enoki Y., Watanabe H., Arake R., Fujimura R., Ishiodori K., Imafuku T., Nishida K., Sugimoto R., Nagao S., Miyamura S. (2017). Potential therapeutic interventions for chronic kidney disease-associated sarcopenia via indoxyl sulfate-induced mitochondrial dysfunction. J. Cachexia Sarcopenia Muscle.

[125] Jheng J.R., Chen Y.S., Ao U.I., Chan D.C., Huang J.W., Hung K.Y., Tarng D.C., Chiang C.K. (2018). The double-edged sword of endoplasmic reticulum stress in uremic sarcopenia through myogenesis perturbation. J. Cachexia Sarcopenia Muscle.

[126] Honda H., Qureshi A.R., Axelsson J., Heimburger O., Suliman M.E., Barany P., Stenvinkel P., Lindholm B. (2007). Obese sarcopenia in patients with end-stage renal disease is associated with inflammation and increased mortality. Am. J. Clin. Nutr..

[127] Yuan J., Watanabe M., Suliman M., Qureshi A.R., Axelsson J., Barany P., Heimburger O., Stenvinkel P., Lindholm B. (2015). Serum hepatocyte growth factor is associated with truncal fat mass and increased mortality in chronic kidney disease stage 5 patients with protein-energy wasting. Nephrol. Dial. Transplant..

[128] Sarkozy M., Kovacs Z.Z.A., Kovacs M.G., Gaspar R., Szucs G., Dux L. (2018). Mechanisms and Modulation of Oxidative/Nitrative Stress in Type 4 Cardio-Renal Syndrome and Renal Sarcopenia. Front. Physiol..

[129] Sato E., Saigusa D., Mishima E., Uchida T., Miura D., Morikawa-Ichinose T., Kisu K., Sekimoto A., Saito R., Oe Y. (2017). Impact of the Oral Adsorbent AST-120 on Organ-Specific Accumulation of Uremic Toxins: LC-MS/MS and MS Imaging Techniques. Toxins (Basel).

[130] Changchien C.Y., Lin Y.H., Cheng Y.C., Chang H.H., Peng Y.S., Chen Y. (2019). Indoxyl sulfate induces myotube atrophy by ROS-ERK and JNK-MAFbx cascades. Chem. Biol. Interact..

[131] Gortan Cappellari G., Semolic A., Ruozi G., Vinci P., Guarnieri G., Bortolotti F., Barbetta D., Zanetti M., Giacca M., Barazzoni R. (2017). Unacylated ghrelin normalizes skeletal muscle oxidative stress and prevents muscle catabolism by enhancing tissue mitophagy in experimental chronic kidney disease. FASEB J..

[132] Wong T.C., Chen Y.T., Wu P.Y., Chen T.W., Chen H.H., Chen T.H., Yang S.H. (2015). Ratio of Dietary n-6/n-3 Polyunsaturated Fatty Acids Independently Related to Muscle Mass Decline in Hemodialysis Patients. PLoS ONE.

[133] Fellstrom B.C., Jardine A.G., Schmieder R.E., Holdaas H., Bannister K., Beutler J., Chae D.W., Chevaile A., Cobbe S.M., Gronhagen-Riska C. (2009). Rosuvastatin and cardiovascular events in patients undergoing hemodialysis. N. Engl. J. Med..

